# Psychological autopsy of suicide—a cross-sectional study

**DOI:** 10.4103/0019-5545.55935

**Published:** 2005

**Authors:** Farooq Ahmed Khan, B. Anand, M. Gowri Devi, K. Krishna Murthy

**Affiliations:** * Senior House Officer, Maudsley Hospital, London, UK; ** Professor, Institute of Mental Health, Hyderabad; *** Professor, Institute of Mental Health, Hyderabad; **** Professor and Head, Institute of Mental Health, Hyderabad

**Keywords:** Psychological autopsy, suicide, psychiatric disorders

## Abstract

**Background:**

Psychological autopsy is the reconstruction of events leading to death. There are few studies on psychological autopsy.

**Aim:**

To understand the profile of suicide completers and find out ways of dealing with it.

**Methods:**

Fifty suicide cases were analysed. Using a semi-structured, self-designed questionnaire, the family, friends and relatives of the deceased were interviewed.

**Results:**

The presence of some type of psychiatric disorder and stressful life events are two important reasons for committing suicide.

**Conclusion:**

Psychological autopsy is a very important tool for assessing the causes and precipitants of suicide. More and more studies in this field are required with a larger sample size for the evaluation of suicides.

## INTRODUCTION

A psychological autopsy is the reconstruction of events leading to death; ascertainment of the circumstances of the death, including suicidal intent; and an in-depth exploration of other significant risk factors for suicide.^1^–^7^ Psychological autopsy is the standard approach to augment the information obtained from a death certificate. Information is gathered through a semi-structured interview of key informants, and discrepancies are resolved by re-interviewing informants and through a case conference using ‘best estimate’ procedures.^8^

The WHO statistics of 2001 on suicide were both surprising and alarming. In 1950, the global suicide rate was 16 per 100,000 population in men and 5 per 100,000 population in women. In 1995, the suicide rate among men went up to 25 per 100,000 population and that among women to 6 per 100,000 population.

A study by Gururaj and Issac shows that the suicide rate in various age groups is as follows: 38 per 100,000 population in the age group of 15–29 years, 34 per 100,000 in those 30–44 years, 18 per 100,000 population in those 45–49 years, 7 per 100,000 population in those ≥60 years.^9^ They also showed that the incidence is higher in men than in women (men 59%, women 41%).

There are few psychological autopsy studies and work in this area is not easy. Right from data collection to completion of the questionnaire, and getting the full and required information from family members, the task is difficult and painstaking. Nevertheless, these types of studies are known to give a comprehensive view of the root of the problem and help us draw various strategies to prevent suicide. Thus, it can be seen that the primary prevention of suicide cannot be realistically planned given the present state of our knowledge. Secondary prevention in individuals who have attempted suicide is, however, encouraging.

### Review of the Literature

Suicide is not a recent phenomenon in most cultures and societies. A lot of work has been done on the subject of suicide both in India and the West. Marked differences were noted in geographical areas, gender, modes used and almost all variables in the field of suicide.

#### Indian studies

Badrinarayana in his study revealed a positive and significant association of depressive illness and suicidal tendency with early parental deprivation, recent bereavement and positive family history of suicidal behaviour.^10^

Banerjee *et al.* studied the vulnerability of Indian women to suicide and found that in their sample two-third of the victims were below 25 years of age.^11^ In women the commonest cause of suicide was a quarrel with the husband and in men it was a quarrel with the parents. Poisoning with insecticides was the most common mode of suicide.

Kar *et al.* worked on adolescent suicide attempters and observed that there has been an alarming increase in the rates of suicide attempts by older children and adolescents.^12^ In their sample, 61.3% were in their late adolescence and females outnumbered males in the ratio of 2.1:1. The most prevalent psychiatric disorder was depression (29%) and all those diagnosed were in late adolescence. Attempters had more stressful life events within 6 months of the attempt in comparison to the control group. In 61% of suicide attempters the mortality was 50% or more.

#### Studies predicting the association of suicide and psychiatric disorders

Unni and Mani studying suicide ideators in a general hospital concluded that 59.74% of people had depression, 9.74% each had substance abuse and psychosis, 7.14% had neurotic disorders, 9.09% had bipolar disorder and 0.65% had normal mental status.^13^ Sixty per cent were housewives and the majority of ideators were in the age group of 16–45 years.

In another study, Sharma noted that 85.4% of the cases were in the age group of 15–34 years.^14^ Among suicide attempters, 53% were female and 52% unmarried. Thirty-two per cent were housewives and 28% were students. A vast majority (74.7%) had consumed organophosphorus compounds. Psychiatric disorders (46.7%), quarrel with the spouse/in-laws (13.4%), quarrel with the parents (12%) and failure in love (10.6%) were some of the most common causes of attempted suicide while no cause could be determined in 14.7% of cases. Jain *et al.* noted similar findings.^15^

#### Western studies

Dyer and Kreitman concluded that while both depression and hopelessness correlate with the degree of suicidal intent as measured on the Suicide Intent Scale, the relationship between depression and suicide intent is dependent on that between hopelessness and suicide intent.^16^

Beck *et al.* found that out of 207 patients hospitalized for suicidal ideation, 14 patients had committed suicide.^17^ Of all the data collected at the time of hospitalization only the Hopelessness Scale and pessimism items of the Beck Depressive Inventory predicted the eventual suicides. A score of 10 or more on the Hopelessness Scale correctly identified 91% of the eventual suicides.

In the study by Shafii *et al.* psychological autopsy of 20 children and adolescents in the age group of 12–19 years who had committed suicide and a matched-pair control group revealed that 85% of the victims and 18% of the control subjects had expressed suicidal ideation.^18^ A statistically significant number of victims had a history of suicidal threat (55%), suicidal attempt (40%), drug or alcohol abuse (70%), antisocial behaviour (70%) or inhibited personality (65%). Suicidal behaviour of parents, relatives and friends, and a parental history of emotional problems and abuse or abusiveness were also significant risk factors for the victim.

Hawton concluded that in the assessment of suicide the relevant factors other than psychiatric disorders are physical illnesses, bereavement, family history of suicide, unemploy-ment and biological factors.^19^

King's work on suicide among mentally ill patients concluded that over 90% of patients were receiving medical care at the time of death, though not all were treated appropriately.^20^ It is important to recognize medical and social risk factors in patients and monitor treatment effectively. Soloff *et al.*^21^ found that the risk factors for suicidal behaviour in patients with borderline personality disorder include old age, prior suicide attempts, antisocial personality disorders, impulsive action and depressed mood but not co-morbid affective disorders, alcoholism or drug use disorder.

Cooper, working on ethical issues regarding psychological autopsy, noted that this method provides detailed information about suicide using various sources including the coroner's report, medical records and information gathered from interviews with key informants.^4^

Isometsa found that psychological autopsy was the most valuable tool for research on completed suicides.^22^ It involves collecting the available information on the deceased via structured interviews of family members, relatives or friends, as well as the attending health care personnel. In addition, information is collected from available health care and psychiatric records, other documents and forensic examination.

The present study aimed to find out the profile of those completing suicide with regard to psychological morbidity, suicidal behaviour in the family, previous suicidal behaviour, present suicidal behaviour and the family's knowledge of suicide.

## METHODS

The study was conducted at the mortuary of the Forensic department of Gandhi General Hospital, Secunderabad. Both the urban and the rural population were included in the study. The families, relatives and friends of the deceased were assessed at the hospital and also at their homes.

### Subjects

Fifty cases of suicide completers were analysed. The deceased of both sexes (42% female and 58% male), aged 15–35 years were included in the study. The family members, relatives and friends were interviewed.

### Procedure

The nature of the study and the questionnaire was fully explained to the family members, relatives and friends. Verbal consent was taken from each individual before the interview.

### Inclusion criteria

The deceased were selected from the cases sent for medical autopsy at Gandhi General Hospital in March 2003 (*n*=50). Retrospective diagnoses of psychiatric disorders were made by questions, which presumably fit into the diagnostic criteria for the particular disorders.

### Tool used

A semi-structured, self-designed questionnaire with all the requirements of a structured interview was used for the psychological autopsy. The details included age, sex, marital status, geographical area, social status, educational level, monthly income, history of psychiatric disorders, history of treatment, duration of consultation with the physician before the attempt. A significant number of studies have reported the importance of stressful life events in suicide and hence we included this in the questionnaire. Details of previous and present suicide attempts, the family's knowledge and awareness of suicide were also assessed. Details about stress related to specific points in time such as examinations/ entrance tests, broken affairs, assaults, insults and guilt; and feelings of remorse, worthlessness, hopelessness, depressed mood, etc. were evaluated. Forensic experts were consulted for their views on the suicide.

## RESULTS

The suicide rate was highest (40%) among the 20–24 years age group, followed by the 25–29 years age group. The sociodemographic characteristics of the subjects are given in Table 1.

**Table 1:** Sociodemographic variables of the suicide completers

Variable	Percentage
Age (in years)	
15–19	20
20–24	40
25–29	22
30–35	18
Locality	
Urban	72
Rural	28
Social status	
High	6
Middle	40
Low	54
Martial status	
Married	48
Unmarried	52
Widowed	0
Divorced	0
Education level	
Graduation and above	20
Secondary education	34
Primary education	12
Illiterate	34
Monthly income (in rupees)	
3000 and above	46
1500–3000	20
500–1500	34

**Fig. 1:**
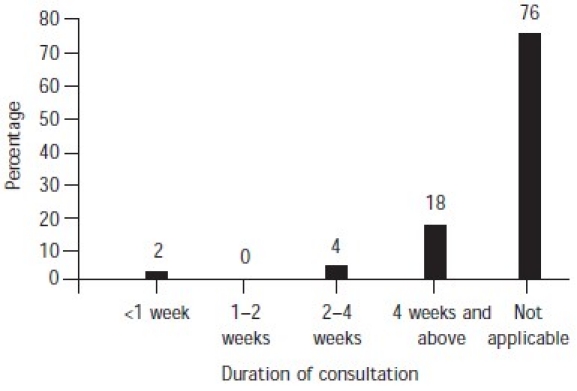
Duration of consultation with physician/psychiatrist before suicide attempt

**Fig. 2:**
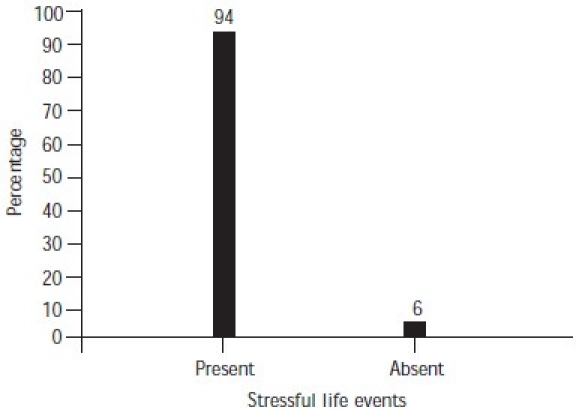
Stressful life events over 6 months–1 year

**Fig. 3:**
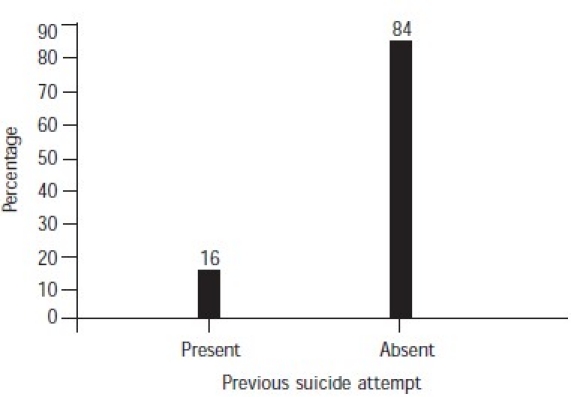
Previous attempt at suicide

The majority of cases did not have any history of taking treatment or medication, psychiatric disorders and substance abuse (Table 2). Among those subjects who had consulted a physician/psychiatrist before their suicide attempt (24%), 18% had been taking treatment for 4 weeks or more, and a very small percentage for less than 4 weeks (Fig. 1). A significant proportion of the subjects (94%) had suffered from stressful life events for 6 months–1 year before the suicide (Fig. 2). In 84% of cases, the subjects had not attempted suicide earlier (Fig. 3).

**Table 2:** History of psychiatric disorders, treatment taken and substance abuse

Result	History of psychiatric disorder	History of treatment/medication	Substance abuse
Present	24	22	18
Absent	76	78	82

**Fig. 4:**
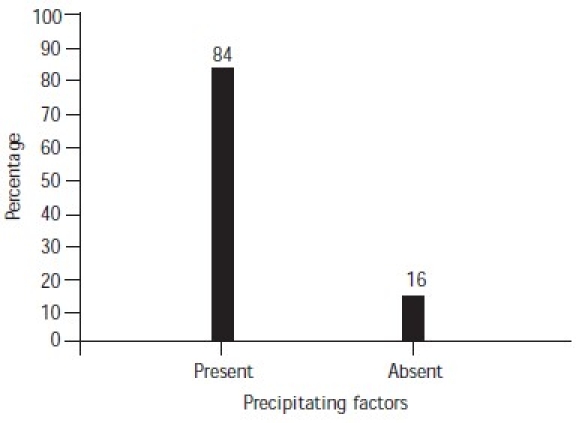
Precipitating factors for suicide

**Fig. 5:**
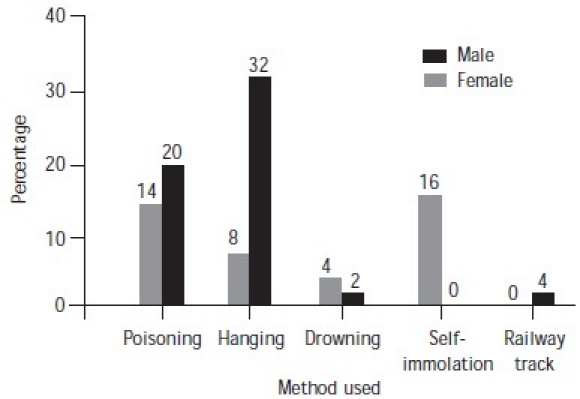
Methods used by males and females

**Fig. 6:**
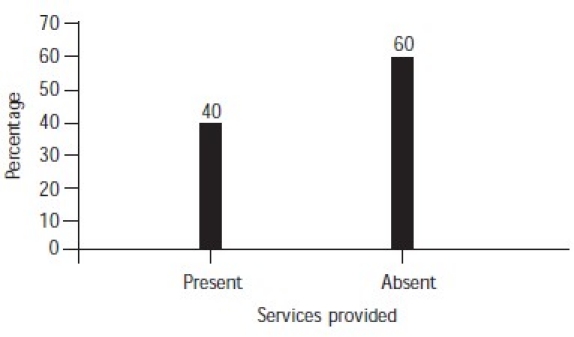
Counselling services provided

Precipitating factors were present in 84% of the subjects (Fig. 4). The most common method used by male suicide completers was hanging and that used by females was self-immolation (Fig. 5). Services had been provided to 40% of the subjects (Fig. 6). Most of the families of subjects (68%) had knowledge of their suicidal tendency, 40% had knowledge of the need for counselling for suicidal patients and 38% knew about psychiatric help for suicidal patients (Table 3).

**Table 3:** Family's knowledge about suicide

	Knowledge of suicidal tendency (%)	Need for counselling suicidal patients (%)	Seeking psychiatric help for suicidal patients (%)
Present	68	40	38
Absent	32	60	62

## DISCUSSION

There have been studies which correlate the age, sex, social background, educational and other variables to ascertain what really triggers the suicidal ideation. The present study demonstrates variability in the sociodemographic pattern, especially in the rural and urban populations. The importance of the family's knowledge about the suicide and its implication on the outcome of the attempt is astounding. Though the psychological autopsy studies are time-consuming, they have their effect in terms of preventive strategies which can be deployed.

### Age and sex

This study found that the suicide rate was the highest in the age group of 20–24 years. Among western studies, importance is given to the prevalence of suicide in high school. An increase in the number of suicides in the past few years among older children and adolescents has been noted.^12^ Another study supports our data that suicide attempts are more common among younger age groups living in urban areas.^23^

### History of psychiatric disorders and treatment

Many studies have supported the fact that major depressive disorder is one of the most common conditions associated with a suicide attempt.^12^,^24^ we found that 12 of the deceased (24%) had some form of psychiatric disorder. Of these, 3 had a psychotic type of illness, most probably paranoid schizophrenia, 5 had major depressive disorder, 2 had bipolar affective disorder (BPAD)—presently depression before the suicide—and 2 had mental retardation.

In one study, 57% were found to have psychiatric illnesses; depression was the most prevalent (37.5%).^15^ In another study suicide ideators were studied in the general hospital set-up and it was found that 59.74% had depression, 9.74% had substance abuse, 9.74% had psychosis, 7.14% had neurotic disorders and 9.09% suffered from bipolar disorder.^13^

All the patients who were suffering from psychiatric disorders in our sample were under treatment from physicians or psychiatrists at the time of committing suicide. Of the 12 people, 9 were under treatment for 4 weeks and more before the attempt. Most of these 9 patients had had consultations 3 months before their attempts. One patient had even consulted the psychiatrist just 2 days before the attempt. This highlights the importance of evaluation of suicidal behaviour by physicians.

### Substance abuse

It was found that 9 of the deceased (18%) had substance abuse. Of these 9 cases, 2 had multiple substance abuse with alcohol and cannabis, and 7 had only alcohol abuse. Unni and Mani^13^ found that substance abuse was present in 9.74% of suicide attempters. Alcohol was found to be an important factor for attempting suicide in 10.46% of patients.^25^ In a western study alcoholism was present in 23% of cases of completed suicide.^19^

### Stressful life events over 6 months to 1 year

A large number of people had significant stressful events. These included economic and occupational factors, the death of significant ones, strained family relations, divorce/ separation, lack of children, broken love affairs, failure in examinations and others. Many of them had more than one stressful event. Of the 47 deceased who had stressful life events, 22 had a single event and 25 had multiple stressful life events.

Economic hardship and strained relations with family members are important stressors. Among young suicide committers, broken love affairs were common. Pressure to secure a seat in Medicine/Engineering tests is also one of the reasons for committing suicide. There are not many studies on educational burden and suicide in the teens which should be focused upon. Kar *et al.* found that attempters of suicide had more stressful life events within 6 months of the attempt.^12^ Interpersonal problems as stressors for suicide have also been pointed out in a few studies.^23^

### Previous attempts

Previous attempts at suicide had been made by 8 of the deceased (16%). Almost all the attempters needed services such as hospitalization. It was found that repeat attempters of suicide did not improve despite treatment and hospitalization (Table 4). Thanki *et al.*^23^ found that interpersonal and financial problems, and major physical illnesses were the most frequent precipitating factors. In another study it was seen that 23.25% of the sample had contemplated before the episode.^25^

**Table 4:** Response of repeat attempters to hospitalization

No. of attempts	Severity of attempt	Precipitating factors	Method used	Response to hospitalization services
1	Severe	—	Poisoning	Improved
1	Severe	Lack of children	Poisoning	Improved
1	Severe	Harassment from the owner of the house	Poisoning	Improved
1	Severe	Financial	Self-immolation	Improved
2	Severe	Quarrel with husband	Drowning	Not improved
	Severe	Quarrel with husband	Hanging	Not improved
1	Severe	Quarrel with husband	Poisoning	Improved
1	Severe	Harassment from in-laws	Poisoning	Improved
4	Severe	—	Poisoning	Not improved
	Severe	—	Poisoning	Not improved
	Severe	—	Hanging	Not improved
	Severe	—	Hanging	Not improved

The commonest method of committing suicide was by hanging (40%) followed by poisoning (34%) mainly with insecticides and occasionally with diazepam or other tranquillizers, self-immolation (16%), drowning (6%) and lying down on the railway tracks (4%). In females the most common mode was self-immolation (16%) followed by poisoning (14%). Hanging was the most common mode of suicide among males (32%) followed by poisoning (20%). An important factor is the presence of a suicide note–24% of our sample had written letters/notes describing the factors responsible for their suicidal behaviour.

Forty per cent of the cases had been hospitalized and almost all were cases of poisoning and a few of self-immolation. Cases of hanging were found dead and services could not be provided to them.

## CONCLUSIONS

We aimed to study the profile of those who had completed suicide on sociodemographic status, psychiatric morbidity, substance abuse, previous suicide attempts, stressful life events responsible for suicide and the family's awareness of the suicidal behaviour. We conclude that psychological autopsy is a very important tool for assessing the causes and precipitants of suicide. It is very difficult to assess the exact reason or pinpoint the cause of suicide. More studies in this field are required with a larger sample size for the evaluation of suicide.

## References

[1] Beskow J, Runeson B, Asgard U (1991). Ethical aspects of psychological autopsy. Acta Psychiatr Scand.

[2] Brent DA, Perper JA, Goldstein CE (1988). Risk factors for adolescent suicide: A comparison of adolescent suicide victims with suicidal inpatients. Arch Gen Psychiatry.

[3] Brent DA, Perper JA, Moritz G (1993). Psychiatric risk factors for adolescent suicide: A case–control study. J Am Acad Child Adolesc Psychiatry.

[4] Cooper J (1999). Ethical issues and their practical application in psychological autopsy study of suicide. J Clin Nursing.

[5] Hawton K, Simkin S, Fagg J (1995). Suicide in Oxford University students, 1976–1990. Br J Psychiatry.

[6] Kelly TM, Mann JJ (1996). Validity of DSM-III-R diagnosis by psychological autopsy: A comparison with clinician ante-mortem diagnosis. Acta Psychiatr Scand.

[7] Velting DM, Shaffer D, Gould MS (1998). Parent–victim agreement in adolescent suicide research. J Am Acad Child Adolesc Psychiatry.

[8] Mitchell RE (1982). Social networks and psychiatric clients: The personal and environmental context. Am J Community Psychol.

[9] Gururaj G, Isaac MK (2001). Epidemiology of suicides in Bangalore.

[10] Badrinarayana A (1980). Study of suicidal risk factors in depressive illness. Indian J Psychiatry.

[11] Banerjee G, Nandi DN, Nandi S (1990). The vulnerability of Indian women to suicide—a field study. Indian J Psychiatry.

[12] Kar N, Das I, Mishra BN (1995). A study of suicide attempts by adolescents. Indian J Psychiatry.

[13] Unni KES, Mani AJ (1996). Suicide ideators in the psychiatric facility of a general hospital—a psychodemographic profile. Indian J Psychiatry.

[14] Sharma RC (1998). Attempted suicide in Himachal Pradesh. Indian J Psychiatry.

[15] Jain V, Singh H, Gupta SC (1999). A study of hopelessness, suicidal intent and depression in cases of attempted suicide. Indian J Psychiatry.

[16] Dyer JA, Kreitman N (1984). Hopelessness, depression and suicidal intent in parasuicide. Br J Psychiatry.

[17] Beck AT, Steer RA, Kovacs M (1985). Hopelessness and eventual suicide: A 10-year prospective study of patients hospitalized with suicidal ideation. Am J Psychiatry.

[18] Shafii M, Carrigan S, Whittinghill JR (1985). Psychological autopsy of completed suicide in children and adolescents. Am J Psychiatry.

[19] Hawton K (1987). Assessment of suicide risk. Br J Psychiatry.

[20] King R (1994). Teaching aboriginal people about suicide awareness and prevention. Addendum to a prior submission to the Parliamentary Standing Committee on Social Issues, Wagga Wagga, August.

[21] Soloff PH, Lis JA, Kelly T (1994). Risk factors for suicidal behavior in borderline personality disorder. Am J Psychiatry.

[22] Isometsa ET (2001). Psychological autopsy studies—a review. Eur Psychiatry.

[23] Thanki G, Mehta A, Chauhan A (1998). Sociodemographic profile and risk assessment in attempted suicide patients in general hospital setting. Indian J Psychiatry.

[24] Gupta SC, Singh H, Trivedi JK (1992). Evaluation of suicidal risk in depressives and schizophrenics: A 2-year follow-up study. Indian J Psychiatry.

[25] Ponnudurai R (1986). Attempted suicide in Madras. Indian J Psychiatry.

